# HPV16-Immortalized Cells from Human Transformation Zone and Endocervix are More Dysplastic than Ectocervical Cells in Organotypic Culture

**DOI:** 10.1038/s41598-018-33865-2

**Published:** 2018-10-18

**Authors:** Han Deng, Eric Hillpot, Sumona Mondal, Kamal K. Khurana, Craig D. Woodworth

**Affiliations:** 1 Department of Biology, Clarkson University, Potsdam, NY United States of America; 2 Department of Mathematics, Clarkson University, Potsdam, NY United States of America; 3 Department of Pathology, SUNY Upstate Medical University, Syracuse, NY USA

## Abstract

A major risk factor for cervical cancer is persistent infection with high-risk human papillomaviruses (HPV) which can cause cervical intraepithelial neoplasia. Greater than 90% of cervical cancers develop in the transformation zone (TZ), a small region of metaplastic squamous epithelium at the squamocolumnar junction between endocervix and ectocervix. However, it is unclear why this region is highly susceptible to malignant progression. We hypothesized that cells from TZ were more susceptible to dysplastic differentiation, a precursor to cervical cancer. We used three-dimensional organotypic culture to compare differentiation of HPV16-immortalized epithelial cell lines derived from ectocervix, TZ, and endocervix. We show that immortal cells from TZ or endocervix form epithelia that are more dysplastic than immortal cells from ectocervix. A higher percentage of immortal cells from TZ and endocervix express the proliferation marker Ki-67 and are positive for phospho-Akt. Immortal cells from TZ and endocervix invade collagen rafts and express increased levels of matrix metalloproteinase-1. Inhibition of MMP-1 or Akt activity blocks invasion. We conclude that HPV16-immortalized cells cultured from TZ or endocervix are more susceptible to dysplastic differentiation, and this might enhance their susceptibility to cervical cancer.

## Introduction

Cervical cancer is a major cause of death in women throughout the world^1^ and the major risk factor for this disease is persistent infection with high-risk HPV types such as HPV16^2^. Most cervical cancers selectively retain and express the HPV E6 and E7 oncogenes, and high-risk HPV16 E6 and E7 proteins can immortalize human cervical epithelial cells^3,4^. Although HPV infections occur frequently in sexually active individuals, the majority are eliminated by the host’s immune system^5^. Two important questions are, “Why do a small subset of high-risk HPV infections progress to cancer and what is unique about these cells?

**Figure 1:**
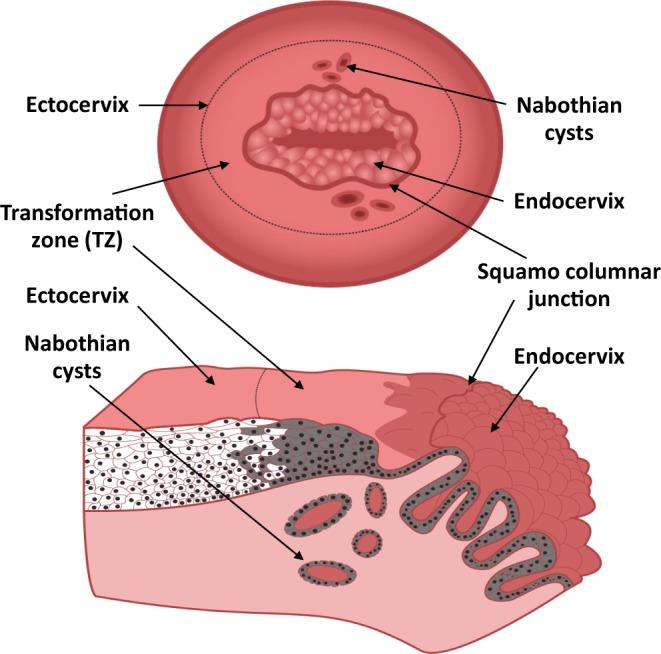
Schematic of the cervical transformation zone. (Top) View of cervix as seen through gynecologist’s speculum showing ectocervix, TZ with Nabothian cysts, and endocervix. (Bottom) Cross section of transformation zone showing columnar epithelium of endocervix and stratified squamous epithelium of TZ and ectocervix. Nabothian cysts form when mucous ducts of endocervix become occluded by overgrowth of stratified squamous epithelium from newly formed TZ. Brown shading illustrates cells derived from endocervical reserve cells.

Almost all cervical cancers arise in a small anatomic area^6^ known as the cervical transformation zone (TZ). This region develops between the secretory columnar epithelium of the endocervix and the stratified squamous epithelium of the ectocervix (Fig. 1). The TZ contains metaplastic squamous cells derived from stem cells (reserve cells) of the endocervix. Although the majority of cervical cancers originate from the TZ, it is unclear why this region is most susceptible to malignant conversion. Several hypotheses have been investigated including the existence of localized immune suppression in this region^7^, increased expression of estrogen receptors on metaplastic epithelial or stromal cells of TZ^8^, increased cell proliferation and unstable differentiation of metaplastic cells^9^, or an increased concentration of stem cells within the TZ^10^.

The epithelium of normal ectocervix and TZ is composed of stratified squamous epithelium formed by continuous movement of cells from the basal to superficial layers. Upward movement is accompanied by cell differentiation, cell flattening and expression of genes for structural proteins such as keratins^11^. Persistent infection by high risk HPVs stimulates aberrant squamous differentiation termed dysplasia or cervical intraepithelial neoplasia (CIN). These dysplastic lesions may persist, regress, or progress in severity to form invasive cancer. Therefore, high grade CIN is a precancerous change with the potential for malignant conversion^12^. The mechanisms by which high-risk HPV causes dysplastic epithelial differentiation have been studied *in vitro* using organotypic cultures^13–16^ or *in vivo* using tissue grafts^17^ or transgenic mouse models^18^.

We recently derived a series of HPV16-immortalized cell lines from human ectocervix, endocervix and TZ^19^. Here, we examine whether immortal cells from TZ are more susceptible to dysplastic epithelial differentiation than cells from ectocervix or endocervix. We cultured HPV16-immortalized cell lines from each cervical region on organotypic cultures composed of collagen rafts. Organotypic cultures provide a three-dimensional model that maintains cell-cell and cell-substrate interactions that are important for cell differentiation^20^. We constructed collagen rafts with either immortal 3T3-J2 mouse cells or primary human cervical stromal cells. We compared the degree of dysplastic epithelial differentiation, cell proliferation and invasion of collagen rafts for immortal cells derived from each cervical region. We found that HPV16-immortalized cells from TZ and endocervix became more dysplastic and invaded collagen rafts more frequently than cell lines from ectocervix.

## Results

### Primary cells from ectocervix, TZ or endocervix undergo stratified squamous differentiation on organotypic culture

**Figure 2:**
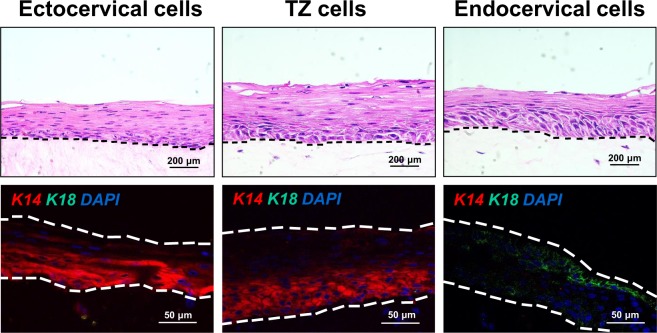
Primary cervical epithelial cells form differentiated stratified squamous epithelia when grown in organotypic culture. (Top row) Normal primary cells were cultured from ectocervix, TZ, or endocervix and maintained for 10 days on collagen rafts containing human cervical stromal cells. Dotted lines indicate position of basement membranes. (Bottom row) Ectocervical, TZ and endocervical cells simultaneously stained for K14 (red), K18 (green) and DAPI (blue).

Before using raft cultures to study dysplastic differentiation, we checked whether our system supported squamous differentiation of normal cervical cells. We maintained primary cultures from each cervical region on collagen rafts containing human cervical stromal cells. We observed that epithelial cells from ectocervix, TZ, or endocervix produced well-differentiated stratified squamous epithelia in organotypic culture (Fig. 2). Cells from ectocervix and TZ expressed keratin 14 (K14) in basal layers but lacked K18. In contrast, endocervical cells expressed K18 but no K14 (Fig. 2). These results confirmed that squamous differentiation and keratin expression in organotypic culture resembled the pattern of differentiation observed *in vivo*^21^. However, the organotypic culture system did not promote columnar secretory differentiation of endocervical cells as occurs *in vivo*^21^.

### HPV16-immortalized epithelial cells from TZ and endocervix become more dysplastic than ectocervical cells

**Figure 3:**
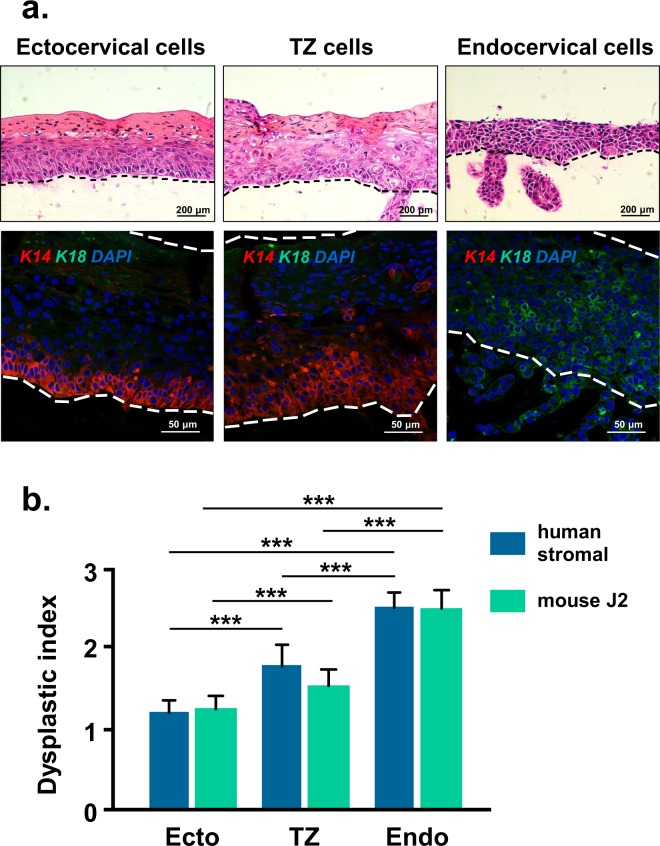
HPV16-immortalized cells from TZ and endocervix are more dysplastic. (**a**) HPV16-immortalized cells derived from ectocervix, TZ, or endocervix were maintained for 10 days on rafts with cervical stromal cells. All cell lines were tested at approximately 80 to 95 population doublings. Dotted lines indicate position of basement membrane and the upper extent of epithelial cells. Upper row of figures were stained with H&E and lower row were simultaneously stained for K14, K18 and DAPI. (**b**) Mean dysplastic index (degree of dysplasia) ± standard error of 24 different HPV16-immortalized cell lines (8 from ectocervix, TZ and endocervix) maintained in organotypic culture on rafts formed with 3T3-J2 mouse cells or human stromal cells. Bars above graph show values that are statistically different (3 asterisks = p < 0.001).

We tested a series of HPV16-immortalized cell lines derived from ectocervix, TZ, and endocervix of eight different patients^19^. We used each cell line at approximately 80 to 95 population doublings or 25 to 30 passages. Cell lines were maintained on collagen rafts containing either immortal 3T3-J2 mouse cells or primary human stromal cells and cultures were assessed for degree of squamous differentiation. We found that HPV16-immortalized cells from TZ and endocervix became more dysplastic than cells from ectocervix; they had variable size and shape, altered mitotic figures, and a larger nucleus to cytoplasm ratio (Fig. 3a). In addition, flattened squamous cells were limited to the upper epithelial layers in TZ rafts or often absent in endocervix rafts. HPV16-immortalized cells from TZ expressed K14 in both basal and suprabasal layers whereas ectocervical cells had K14 in mainly the basal layers. Endocervical cells expressed only K18.

We examined the dysplastic index of 24 different HPV16-immortalized cell lines (8 from ectocervix, TZ and endocervix) and found that cells from TZ and endocervix had significantly more dysplastic differentiation than ectocervical cells (Fig. 3b and Supplementary Table S1). However, the degree of dysplastic differentiation differed in cell lines derived from different patients. We detected no significant difference in the dysplastic index of HPV16-immortalized cell lines that were grown on rafts with 3T3-J2 mouse cells versus human stromal cells (Fig. 3b).

### HPV16-immortalized cells from TZ and endocervix have higher levels of Ki-67 and phospho-Akt

**Figure 4:**
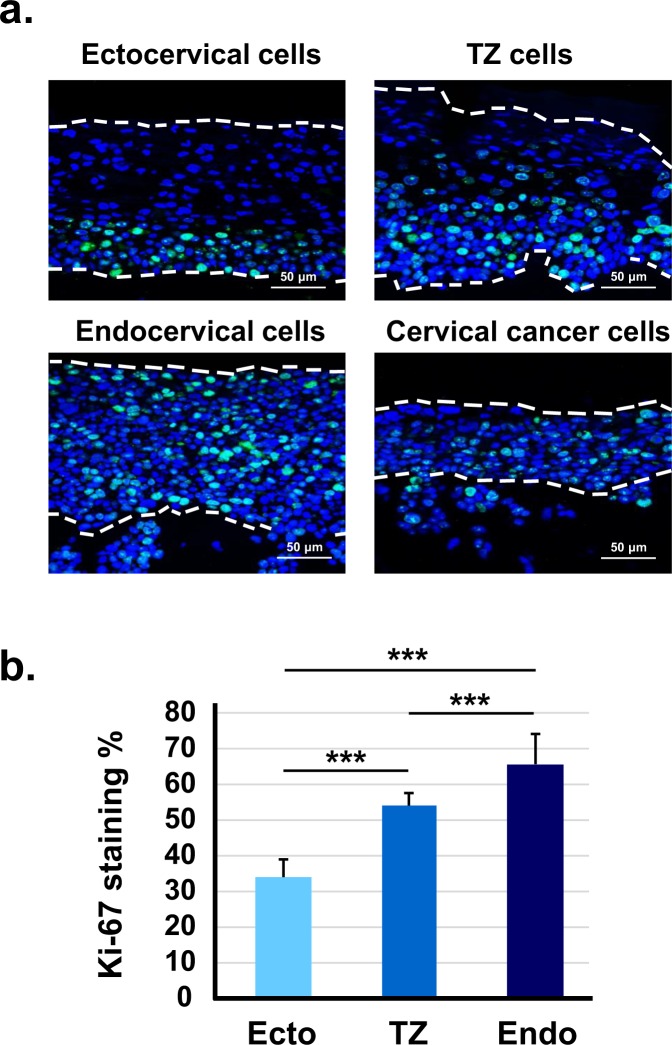
HPV16-immortalized cells from TZ and endocervix have more Ki-67 positive cells. (**a**) HPV16-immortalized cell lines derived from ectocervix, TZ, or endocervix were maintained for 10 days on collagen rafts with cervical stromal cells and then stained with DAPI to visualize cell nuclei and Ki-67 antibody to show proliferating cells. Cervical cancer cells served as a positive control for cell proliferation. Dotted lines indicate position of basement membrane and upper extent of epithelial cells. (**b**) Mean percentage of Ki-67 positive cells ± standard error of 12 different HPV16-immortalized cell lines (4 from ectocervix, TZ and endocervix) maintained in organotypic culture on rafts formed with cervical stromal cells. Bars above graph show values that are statistically different (3 asterisks = p < 0.001).

The Ki-67 protein is a marker for cell proliferation^22^. We stained organotypic cultures with an antibody to Ki-67 and compared the percentage of proliferating cells from ectocervix, TZ, or endocervix. We found that rafts containing immortal cells from TZ and endocervix contained more Ki-67-positive cells and that they occurred throughout the epithelial layers (Fig. 4a,b). In contrast, immortal ectocervical cells had fewer Ki-67-positive cells, which were mainly in the basal epithelial layer.

**Figure 5:**
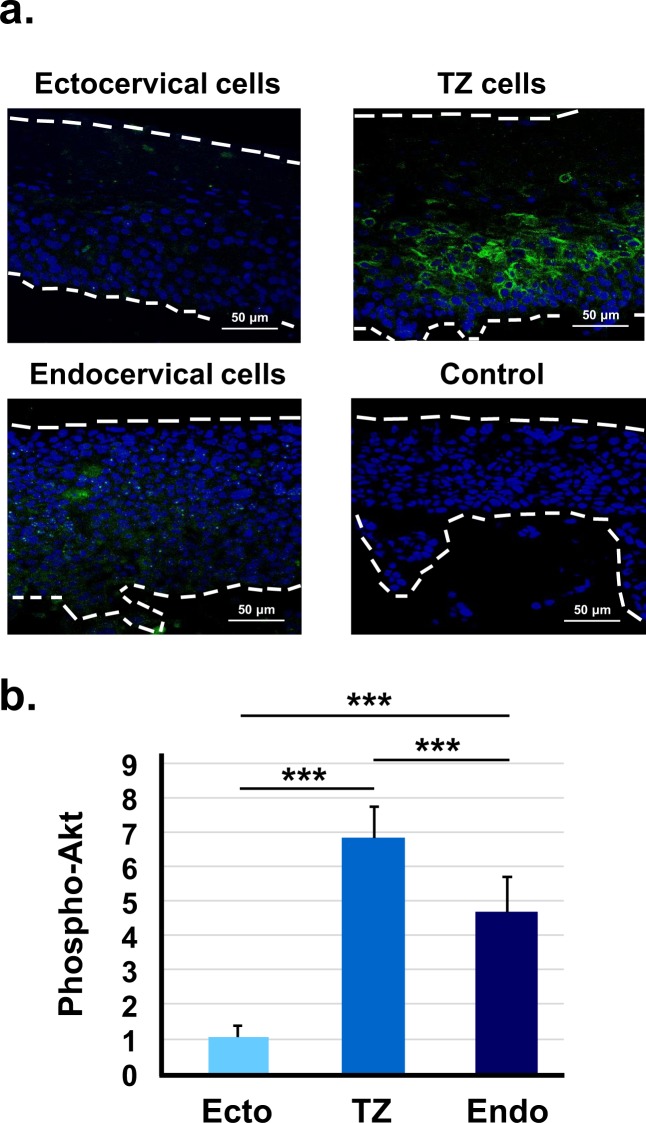
HPV16-immortalized cells from TZ and endocervix express more phospho-Akt. (**a**) HPV16-immortalized cell lines from ectocervix, TZ, or endocervix were maintained for 10 days on collagen rafts with cervical stromal cells and then stained with fluorescent antibody to phospho-Akt. Control indicates cells that were stained with an irrelevant antibody. Dotted lines indicate the position of the basement membrane and the upper extent of epithelial cells. (**b**) Mean percentage of phospho-Akt positive cells ± standard error of nine different HPV16-immortalized cell lines (3 from ectocervix, TZ and endocervix). Bars show values that are statistically different (3 asterisks = p < 0.001).

The phosphatidylinositol 3-kinase (PI3K)/Akt pathway is activated by HPV16 E7^23^ and is often upregulated during cervical dysplasia and carcinogenesis^24,25^. We compared phospho-Akt activation in raft cultures of immortal cells from each cervical region to examine whether it correlated to dysplastic differentiation. We found that immortal cells from TZ and endocervix had more phospho-Akt staining than cells from ectocervix (Fig. 5a,b). Therefore, increased activity of Akt kinase in HPV16-immortalized cells from TZ and endocervix might contribute to increased cell proliferation and dysplastic differentiation.

### HPV16-immortalized epithelial cells from TZ and endocervix invade the stroma of raft cultures and express higher levels of activated matrix metalloproteinase-1 (MMP-1)

**Figure 6:**
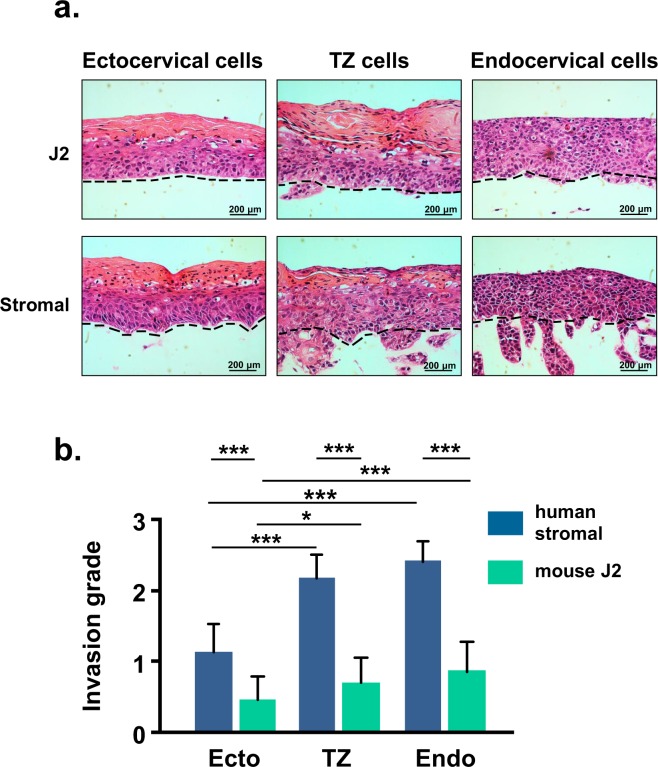
HPV16-immortalized cells from TZ and endocervix are more invasive. (**a**) HPV16-immortalized cells derived from ectocervix, TZ, or endocervix (all 85 population doublings) were grown for 10 days on rafts with either 3T3-J2 mouse cells or human stromal cells. Dotted lines indicate position of basement membranes. (**b**) Mean invasion index (degree of invasion into collagen raft) ± standard error of 24 different HPV16-immortalized cell lines (8 from ectocervix, TZ and endocervix) maintained in organotypic culture on rafts formed with 3T3-J2 cells or human stromal cells. Bars show values that are statistically different (3 asterisks = p < 0.001).

The hallmark of cancer is the ability to invade into the underlying stroma^26^. Although HPV16-immortalized human cells are not tumorigenic when transplanted to nude mice^27^, we noticed that in organotypic culture, immortal cells from TZ and endocervix invaded into the collagen raft (Fig. 6a). In contrast, immortal cells from ectocervix rarely invaded. We examined the invasion grade of 24 different HPV16-immortalized cell lines (8 from ectocervix, TZ and endocervix) and found that cells from TZ and endocervix had significantly more invasion than ectocervical cells (Fig. 6b and Supplementary Table S2). Of particular interest, the type of stromal cells used to construct rafts significantly influenced invasion. Invasion was much greater in rafts that contained human cervical stromal cells compared to rafts that had 3T3-J2 mouse cells (Fig. 6b).

**Figure 7:**
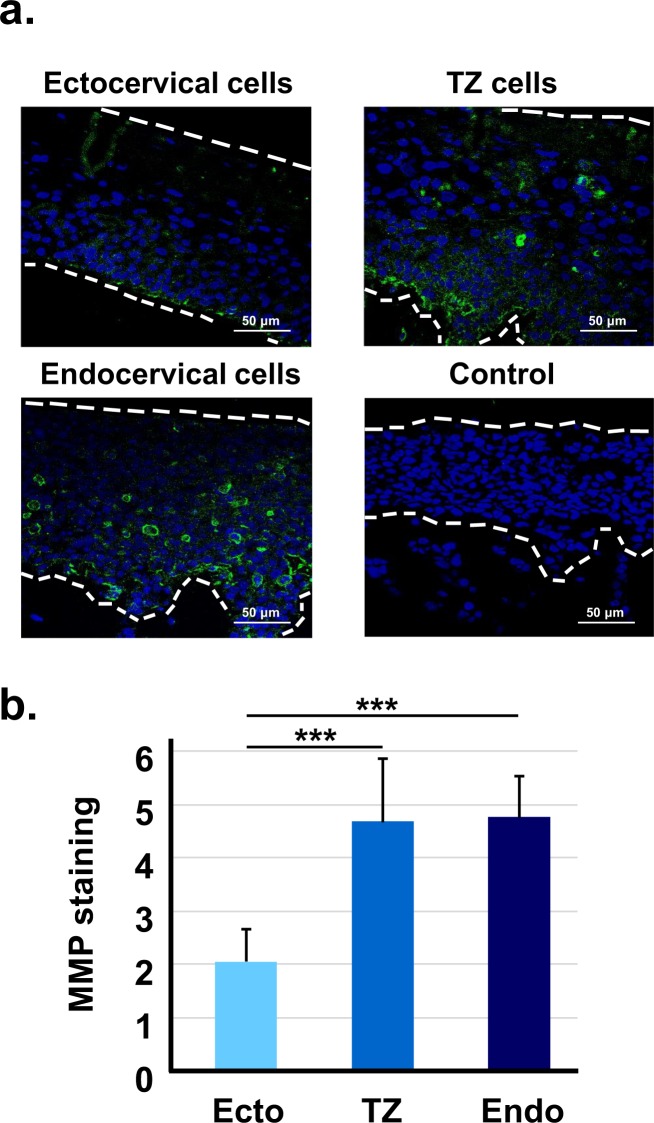
HPV16-immortalized cells from TZ and endocervix express higher levels of MMP-1. (**a**) HPV16-immortalized cells from ectocervix, TZ, or endocervix were maintained for 10 days on collagen rafts with cervical stromal cells, and then stained with fluorescent antibody to MMP-1. Control indicates cells stained with an irrelevant antibody. Dotted lines indicate the position of the basement membrane and the upper extent of epithelial cells. (**b**) Mean percentage of MMP-1 cells ± standard error of nine different HPV16-immortalized cell lines (3 from ectocervix, TZ and endocervix). Bars show values that are statistically different (3 asterisks = p < 0.001).

Increased activation of MMP-1, which digests collagen types I, II, and III, occurs during cervical carcinogenesis^28^ and may contribute to invasion^29^. We compared staining for MMP-1 in organotypic cultures of immortal cells from each cervical region. We found that immortal cells from TZ and endocervix showed significantly more MMP-1 staining than cells from ectocervix (Fig. 7a,b). Thus, increased production of MMP-1 in immortal cells from TZ and endocervix correlated with invasion.

### Inhibition of MMP-1 or Akt kinase blocks invasion of HPV16-immortalized cells from TZ

**Figure 8:**
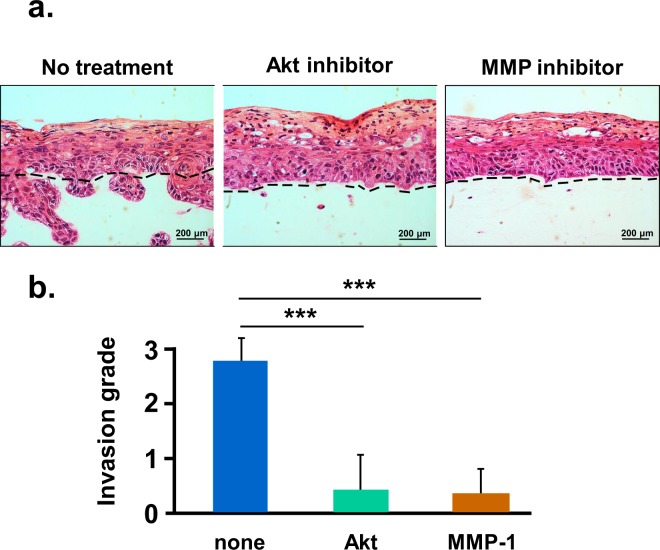
Inhibition of Akt or MMP-1 blocks invasion of HPV16-immortalized cells. (**a**) HPV16-immortalized TZ cells were seeded on collagen rafts with cervical stromal cells and then treated for 10 days with the Akt inhibitor SC 66 (2 µg/ml) or the MMP inhibitor GM6001 (10 μM) in DMSO. Untreated cultures received only DMSO. Dotted lines indicate position of basement membranes. (**b**) Mean invasion index ± standard error of three organotypic cultures. Bars show values that are statistically different (3 asterisks = p < 0.001).

We used inhibitors of MMP-1 or Akt to directly test whether invasion of HPV16-immortalized cervical cells was due to increased MMP-1 or phospho-Akt activity. Collagen rafts were treated with the MMP inhibitor GM6001 (10 μM) or the Akt inhibitor SC 66 (2 µg/ml) in DMSO. Untreated cultures received only DMSO. In untreated rafts, HPV16-immortalized TZ cells invaded the collagen substrate, as expected. However, little or no invasion occurred in rafts treated with either MMP-1 or Akt inhibitors (Fig. 8a). Each inhibitor significantly reduced the number of invading epithelial cells (Fig. 8b). MMP1 and AKT inhibitors did not affect proliferation of epithelial cells in raft cultures when used at the lowest dose that blocked invasion. These results suggest that invasion was dependent on MMP-1 and Akt activity.

## Discussion

Most cervical cancers develop within a narrow region called the TZ, but it is unclear why these cells are highly susceptible to carcinogenesis. Cervical intraepithelial neoplasia (CIN) is a precursor to cervical cancer, and the grade or severity of dysplasia varies from low (CIN1) to high (CIN3). Grading is based on the extent to which abnormal cells occur throughout the epithelium. For example, CIN1 has abnormal cells in the lower third of the epithelium whereas CIN3 has abnormal cells throughout the entire thickness. We hypothesized that HPV16-immortalized cells from TZ would become more dysplastic than cells from ectocervix or endocervix. To test this, we examined eight groups of HPV16-immortalized cell lines derived from ectocervix, endocervix or TZ (24 cell lines) in organotypic culture. We found that immortalized cells from TZ and endocervix became more dysplastic than immortal ectocervical cells. They had greater Ki-67 staining and they invaded into collagen rafts more than cells from ectocervix. Thus, the increased susceptibility of TZ and endocervical cells to develop severe dysplasia might contribute to progression to cervical cancer.

We also grew primary cultures of normal cervical epithelial cells on collagen rafts, and cells from each region formed well-differentiated epithelia. This confirmed that that our methods for organotypic culture were technically sound and that our results were similar to previous studies^14,20^. Immunofluorescence staining showed that primary cells from TZ, ectocervix or endocervix expressed the pattern of keratins (K14 and K18) observed *in vivo*, confirming their region of origin. However, endocervical cells, which normally undergo columnar differentiation *in vivo*, formed stratified squamous epithelia on collagen rafts. Thus, the raft system did not support normal endocervical differentiation. Endocervical cells might respond better or in a different manner in a spheroid type culture system^30^. Raft culture of endocervical cells may recapitulate the process of squamous metaplasia that occurs *in vivo* within the cervical TZ. Both TZ and endocervical cells arise from a common progenitor, endocervical reserve cells^10^, which might explain our unexpected observation that HPV16-immortalized cells from both TZ and endocervix developed severe dysplastic differentiation.

In contrast to normal cells, HPV16-immortalized cells developed dysplastic differentiation resembling CIN1 to CIN3. Our results agree with previous observations that HPV-immortal cells become dysplastic in raft culture or after transplantation in nude mice^14,15,17^. When we tested very low passage cells (35 to 50 population doublings or approximately 10 to 15 passages) that may not have completely emerged from crisis prior to immortalization, they formed thin epithelia that grew slowly. Therefore, we focused on higher passage cells (85 to 95 population doublings or about 25 to 30 passages) for all experiments. These passage numbers are comparable to other studies that examined differentiation of HPV-immortalized cells^14,15,17^. Our work showed that HPV16-immortalized cells from TZ and endocervix became more dysplastic than cells from ectocervix. Because we examined only HPV16, we cannot generalize our results to other high-risk types. During squamous metaplasia, the TZ develops from endocervical reserve cells. If reserve cells are more susceptible to dysplastic differentiation, it is reasonable that immortalized endocervical cells would also be more dysplastic. In a previous *in vivo* transplantation study, HPV16-immortalized endocervical cells developed lesions that resembled carcinoma *in situ* while their ectocervical counterparts formed mildly dysplastic lesions^31^. Our results agree with these findings and identify a greater susceptibility of immortal TZ and endocervical cells for dysplastic progression.

HPV16-immortalized cell lines used for this work have been characterized previously^19^. Most were derived by infection with retroviruses encoding HPV16 E6/E7 genes (18 lines from 6 patients) and contain integrated rather than episomal genomes. Levels of HPV16 E6 and E7 RNAs were similar in cells derived from ectocervix, TZ or endocervix. We examined several biomarkers (in addition to K14 and K18) to identify the origin of the cells. Recent studies describe a discrete population of squamocolumnar junction cells (SC) that have a specific gene expression profile and that may be progenitors for cervical cancer^32^. We detected one of these proteins (MMP-7) in 9 to 13% of cells cultured from TZ or endocervix^19^, but MMP-7 was also expressed at a low level in cells from ectocervix. Cytokeratin 17 (K17) and p63 are two additional markers for reserve cells of TZ and endocervix^21^. We detected K17 in 13 to 25% of cells cultured from TZ or endocervix but not ectocervix^19^. However, p63 was expressed in 5 to 10% of cells cultured from each cervical region^19^. This suggests that cell isolation and cell culture medium might alter the SC gene expression pattern seen *in vivo*. Thus, it might be difficult to relate expression of specific SC markers *in vitro* to their pattern of expression in the cervix. It is unclear whether our HPV16-immortalized cells from TZ or endocervix arose from SC cells. It would be interesting to isolate and purify SC cells from normal cervical tissue and directly test their response to HPV16 in cell immortalization assays or the differentiation of HPV-immortalized squamocolumnar cells when grown on raft cultures.

HPV16 oncoproteins deregulate multiple cell pathways and promote invasion in organotypic culture. For example, HPV16 E6 and E7 proteins can induce invasion by repressing insulin-like growth factor binding protein^33^. Most studies use immortal 3T3-J2 mouse fibroblasts to construct collagen rafts. However, mouse cells might not be optimal for studying human cancer. Here, we compared collagen rafts constructed with either human cervical stromal cells or 3T3-J2 mouse cells. There was no difference in dysplastic differentiation between rafts with stromal or 3T3-J2 cells, but stromal cells promoted much more epithelial invasion. Stromal-epithelial interactions regulate epithelial growth and invasion in different ways^34^. For example, stromal estrogen receptors contribute to cervical carcinogenesis in transgenic mouse models^35,36^. Therefore, organotypic cultures with human cervical stromal cells might provide a better model (relative to 3T3-J2 cells) of the *in vivo* environment that contributes to cervical carcinogenesis. We used pooled cultures of stromal cells from several patients using tissue from all 3 cervical regions. In the future, it might be interesting to test stromal cells from each cervical region to examine whether there are region-specific paracrine effects on epithelial cells.

We found that HPV16-immortalized cell lines from TZ and endocervix expressed more Ki-67, a marker of cell proliferation. The Ki-67 antigen increases during progressive stages of CIN *in vivo*^37^. Additionally, HPV16-immortalized cells from TZ and endocervix expressed more phospho-Akt and MMP-1. The pAkt pathway becomes activated in many cancers^38^, and increased phospho Akt has been correlated with poor prognosis in cervical cancer^39,40^. Therefore, AKT activation may promote dysplastic differentiation and invasion in raft cultures. MMP-1 is associated with epithelial to mesenchyme transition, a process that promotes invasion and metastasis^28,29^. We found that inhibition of either MMP-1 or Akt kinase strongly reduced invasion of HPV16-immortalized cells. Previous work showed the same response for cervical cancer cells^28^. MMP-1 expression is increased by epidermal growth factor via several signal pathways including MAPK/ERK, STAT3 and c-jun^41–44^. Thus, MMP-1 activation might contribute to enhanced invasion of TZ and endocervical cells.

Overall, these experiments showed that HPV16-immortalized cells from TZ and endocervix were more dysplastic and invaded the underlying stroma when grown in organotypic culture. Invasion (but not dysplasia) was stimulated when immortal cells were maintained with human stromal cells rather that mouse 3T3-J2 cells. The invasive and dysplastic cells showed increased activation of Akt kinase and higher expression of MMP-1. Pharmacologic inhibition of each pathway blocked invasion, suggesting that Akt and MMP-1 are important for progression to malignancy.

## Methods

### Cell culture

Primary human cervical epithelial cells and HPV16-immortalized cell lines^19^ were derived and maintained in Keratinocyte Serum-Free Medium (KSFM, GIBCO) containing antibiotics. Primary epithelial cultures were isolated from human cervical tissue in serum-free medium as described^45^. In some experiments, epithelial cells were isolated as cocultures with stromal cells in DMEM supplemented with 10% fetal bovine serum^46^, and cocultured cervical cells were immortalized with HPV16. Serum-free or serum-containing media worked equally well to immortalize cells from ectocervix, transformation zone and endocervix^19^. All tissues were purchased from the Cooperative Human Tissue Network (CHTN) and informed consent of each patient was obtained by CHTN. Tissue samples had no patient identifiers and samples were obtained previously for other purposes. Thus, our experiments were exempt from Institutional Review Board approval by Clarkson University. Human stromal cells were isolated from small fragments (1 × 1 mm) of cervical stromal tissue by digestion with collagenase (0.15% in DMEM w/o serum) at 37 °C for 1 hour. The cell digest was centrifuged at low speed and cells were suspended in DMEM (GIBCO) with 10% FBS (Atlanta Biologicals) plus antibiotics. Stromal cells were isolated and pooled from several patients using tissue from all 3 cervical regions. We froze pooled cultures and used them in all experiments to reduce variability. The mouse 3T3-J2 cell line was purchased from Kerafast and cultured in DMEM with 10% FBS plus antibiotics.

### Organotypic culture

Organotypic culture was performed as previously described^47^. However, in these experiments we omitted epidermal growth factor (EGF) and reduced the concentration of fetal bovine serum to 5%. Collagen rafts were made in 6-well cell culture plates (Corning). Six collagen rafts were formed by mixing 2.5 ml of rat tail collagen I (Millipore), 8 ml of DMEM with 10% FBS (Atlanta Biologicals), 2 ml of DMEM with casein (5 mg/ml), 1 × 10^6^ of 3T3-J2 cells or mitomycin C-treated human stromal cells, and 0.5 ml of 0.1 M NaOH. Two ml of this mixture was aliquoted to each well and incubated at 37 °C for 2 days to allow contraction of rafts before 1 × 10^6^ cervical epithelial cells were allowed to attach to the top of each raft (for 2 hours). All HPV16-immortalized cervical cells were tested at approximately 80 to 95 population doublings or about 25 to 30 passages. Rafts were submerged in raft medium^48^ (1:3 mixture of F12/DMEM medium with 5% FBS plus Supplements) for 48 hours before being raised onto stainless steel grids in a 100 mm cell culture dish to form an air-liquid interface for epithelial cells. The medium was changed every 2 days and cultures were fixed after 10 days.

### Grading of dysplastic differentiation and invasion

The system used to grade CIN in clinical lesions was adapted to grade dysplastic differentiation in raft culture. Cultures were graded as 0 = normal stratified squamous differentiation, 1 = mildly dysplastic with 1/3 thickness of abnormal cells, 2 = moderately dysplastic with 2/3 thickness of abnormal cells, and 3 = severely dysplastic with full thickness of abnormal cells. Abnormal cells were identified based on altered size and shape, abnormal mitotic figures, and reduced cytoplasm to nucleus ratio. Three independent slides were used for grading each different cell line. A total of 30 visual sections (800 μm each) were graded and the mean ± standard error was determined. A pathologist reviewed the grading scheme for dysplastic differentiation and checked results to determine if there were differences in dysplasia from each source of cells. Cell invasion was graded by counting the number of invasive cells: 0 = normal (0 to 1 invasive cell colony), 1 = mild invasion (2 to 4 invasive cell colonies), 2 = moderate invasion (5 to 10 invasive cell colonies), and 3 = severe invasion (more than 10 invasive cell colonies). Three independent slides were used for grading each different cell line. A total of visual 30 sections (800 μm each) were graded and the mean ± standard error was determined.

### Treatment with MMP or Akt Inhibitors

We seeded HPV16-immortalized cells from TZ on collagen rafts containing human cervical stromal cells and then raised rafts on steel mesh grids at the air-liquid interface. On the first day of raft culture, 2 µg/ml Akt inhibitor SC 66 (Tocris Bioscience) or 10 μM MMP inhibitor GM6001 (EMD Millipore™) was added to raft culture medium in 0.1% DMSO as described^49,50^. Raft medium was changed every two days and fresh inhibitor was added. The same amount of DMSO with no inhibitor was added to raft cultures as the untreated controls.

### Immunofluorescence staining

We fixed raft cultures in 4% neutral buffered formalin and then embedded in paraffin for sectioning and hematoxylin and eosin (H&E) staining. Primary rabbit monoclonal antibodies to keratin 14 (ab51054, Abcam) and Ki-67 (ab16667, Abcam) and primary mouse monoclonal antibodies to K18 (ab7797, Abcam), pAkt1 (sc-293125, Santa Cruz) and MMP-1 (sc-21731, Santa Cruz) were used for immunohistochemistry on unstained sections. Secondary donkey anti-mouse antibody (ab150105, Abcam) with Alexa 488 tag and secondary goat anti-rabbit antibody (ab150080, Abcam) with an Alexa 594 tag were used for visualization. Negative controls consisted of irrelevant primary antibodies including rabbit monoclonal isotype control (ab172730, Abcam) and mouse monoclonal isotype control (ab170190, Abcam). The gain and exposure time of the fluorescence microscope were adjusted to show positively stained cells while negative control cells had no staining. Staining percentage was calculated by dividing the positively stained cell number by the total cell number. Staining intensity was measured by ImageJ and the negative control was used to subtract background.

### Statistical analysis

Parametric statistical analysis was performed using one-way analysis of variance (ANOVA) and Tukey’s multiple comparison test for pairwise comparisons with IBM SPSS software version 24. Non-parametric statistical analysis was performed using Kruskal-Wallis test and Dunn-Bonferroni tests for pairwise comparisons with IBM SPSS software version 24. A calculation of two-tailed p value less than 0.05 was considered significantly different. In the “Nonparametric Tests” function, “Kruskal-Wallis 1-way ANOVA (k samples)” was chosen and “All pairwise” comparisons was set. Comparisons were performed to compare the difference between different cervical regions.

## Electronic supplementary material

Supplemental data

## Data Availability

All data generated or analyzed during this study are included in this published article (and its Supplementary Information files).
