# Supplementary material for: HPV16-Immortalized Cells from Human Transformation Zone and Endocervix are More Dysplastic than Ectocervical Cells in Organotypic Culture
Source: Sci Rep. 2018 Oct 18;8:15402. doi: 10.1038/s41598-018-33865-2 (PMC6194146; doi:10.1038/s41598-018-33865-2)
Supplement: Supplementary file 1 — Supplemental data [file 41598_2018_33865_MOESM1_ESM.pdf]

S1 Table. Raw data of dysplastic differentiation with stromal and J2 rafts

		Dysplastic grade		
**Epithelial cell**	**Raft**	**Ecto**	**TZ**	**Endo**
**CX16-RV2**	Stromal	1.20 ± 0.23	1.67 ± 0.27	3.00 ± 0.00
	J2	1.23 ± 0.25	1.80 ± 0.23	3.00 ± 0.00
**CX16-RV3**	Stromal	1.30 ± 0.27	2.70 ± 0.27	2.63 ± 0.28
	J2	1.23 ± 0.25	1.57 ± 0.29	2.57 ± 0.29
**CX16-RV6**	Stromal	1.03 ± 0.11	1.57 ± 0.29	1.70 ± 0.27
	J2	1.13 ± 0.20	1.50 ± 0.29	1.40 ± 0.29
**CX16-RV7**	Stromal	1.07 ± 0.14	1.07 ± 0.14	2.73 ± 0.26
	J2	1.13 ± 0.20	1.13 ± 0.20	2.87 ± 0.20
**CX16-RV8**	Stromal	1.77 ± 0.24	1.97 ± 0.10	2.00 ± 0.00
	J2	1.53 ± 0.29	1.77 ± 0.24	2.57 ± 0.29
**CX16-RV9**	Stromal	1.10 ± 0.17	1.43 ± 0.29	2.23 ± 0.24
	J2	1.13 ± 0.20	1.17 ± 0.22	1.73 ± 0.30
**16-8**	Stromal	1.10 ± 0.18	2.87 ± 0.20	2.83 ± 0.22
	J2	1.10 ± 0.18	2.30 ± 0.27	2.90 ± 0.18
**16-9**	Stromal	1.17 ± 0.22	1.07 ± 0.15	2.97 ± 0.11
	J2	1.60 ± 0.29	1.10 ± 0.18	3.00 ± 0.00
**Mean**	Stromal	1.22 ± 0.15	1.79 ± 0.26	2.51 ± 0.20
	J2	1.26 ± 0.16	1.54 ± 0.20	2.50 ± 0.24

*** Data shown is mean of epithelial dysplastic differentiation grading on 3 independent rafts ± standard error.**

S2 Table. Raw data of invasion grading with stromal and J2 rafts

		Invasion grade		
**Epithelial cell**	**Raft**	**Ecto**	**TZ**	**Endo**
**CX16-RV2**	Stromal	0.20 ± 0.23	1.30 ± 0.41	2.47 ± 0.33
	J2	0.00 ± 0.00	0.10 ± 0.18	0.10 ± 0.18
**CX16-RV3**	Stromal	0.00 ± 0.00	1.10 ± 0.46	1.00 ± 0.45
	J2	0.03 ± 0.11	0.13 ± 0.20	0.13 ± 0.20
**CX16-RV6**	Stromal	0.10 ± 0.18	2.80 ± 0.23	2.30 ± 0.27
	J2	0.17 ± 0.22	2.33 ± 0.44	1.83 ± 0.37
**CX16-RV7**	Stromal	1.90 ± 0.38	1.77 ± 0.51	2.83 ± 0.22
	J2	0.00 ± 0.00	0.00 ± 0.00	0.00 ± 0.00
**CX16-RV8**	Stromal	2.23 ± 0.41	2.83 ± 0.22	2.47 ± 0.47
	J2	0.00 ± 0.00	0.20 ± 0.23	0.10 ± 0.17
**CX16-RV9**	Stromal	1.57 ± 0.36	2.57 ± 0.32	2.97 ± 0.10
	J2	0.00 ± 0.00	0.00 ± 0.00	0.00 ± 0.00
**16-8**	Stromal	0.23 ± 0.25	2.83 ± 0.22	2.70 ± 0.27
	J2	0.63 ± 0.28	0.67 ± 0.32	2.07 ± 0.40
**16-9**	Stromal	2.83 ± 0.22	2.27 ± 0.40	2.53 ± 0.30
	J2	2.83 ± 0.22	2.13 ± 0.37	2.67 ± 0.32
**Mean**	Stromal	1.13 ± 0.41	2.18 ± 0.32	2.41 ± 0.28
	J2	0.46 ± 0.34	0.70 ± 0.36	0.86 ± 0.40

*** Data shown is mean of epithelial invasion grading on 3 independent rafts ± standard error.**
